# Ponseti Casting vs. Soft Tissue Release for the Initial Treatment of Non-idiopathic Clubfoot

**DOI:** 10.3389/fsurg.2021.668334

**Published:** 2021-05-28

**Authors:** Jonathan Abraham, Jon Cooper Wall, Michel Diab, Cody Beaver

**Affiliations:** Department of Orthopaedic Surgery, Texas Tech University Health Science Center, Lubbock, TX, United States

**Keywords:** Ponseti casting, non-idiopathic clubfeet, arthrogryposis, spina bifida, neuromuscular, soft tissue release

## Abstract

**Purpose:** Ponseti casting has universally been accepted as the gold standard for treatment of idiopathic clubfoot. Conversely, primary treatment for non-idiopathic clubfoot has not been established. The purpose of this study is to compare treatment outcomes following primary soft tissue release (STR) and Ponseti casting of non-idiopathic clubfoot.

**Methods:** An IRB-approved retrospective study of patients treated for non-idiopathic clubfoot between 2005 and 2020 was conducted. Patients were included if they began treatment before the age of 2 and had at least 1 year of follow up. Patients were placed into either the STR group or Ponseti group and variables of interest were documented including reoccurrence of deformity, number of surgeries performed, type of surgeries performed, anesthesia time, and surgery time. Data was analyzed using Mann-Whitney U test for continuous variables.

**Results:** A total of 33 children with 57 neuromuscular/syndromic clubfoot were identified of which 9 (15 feet) were treated with STR and 24 (42 feet) were treated with Ponseti casting. Average anesthesia and surgery time were found to be 291 and 179 min, respectively, for the STR group, and 113 and 67 min for the Ponseti group. The difference in operating time was determined to be significant (*p* = 0.02, *p* = 0.01). Patients treated with STR were found to have significantly more surgeries performed over the course of treatment than those treated with Ponseti casting (*p* = 0.001) with an average of 4.2 surgeries in the STR group and 1.5 surgeries in the Ponseti group. Extracapsular procedures were performed in 100% of the STR group and 97.6% of the Ponseti group (*p* = 0.55). Intracapsular procedures were performed in 100% of the STR group and 50% of the Ponseti group (*p* = 0.001).

**Conclusion:** The Ponseti method should serve as the primary approach in the initial treatment of non-idiopathic clubfoot as it can reduce the risk of future invasive intracapsular surgery and shorten anesthesia and surgery times when surgical treatment is necessary.

**Level of Evidence:** Level III retrospective case control study.

## Background

The Ponseti method is accepted as the standard approach within our region in treating idiopathic clubfoot. This technique has shown improved outcomes with a drop in surgical intervention from 72 to 12% and is currently utilized by a large majority of pediatric orthopedic surgeons in North America (1, 2).

Unlike idiopathic clubfoot, non-idiopathic clubfoot has yet to accept a standard approach in treatment. Non-idiopathic clubfoot refers to patients who develop clubfoot secondary to an underlying condition with the most common being spina bifida and arthrogryposis (3). These deformities are more rigid and resistant to treatment compared to idiopathic clubfoot. Traditionally, non-idiopathic clubfoot has been treated surgically, most often with soft tissue release (STR) and serial casting (4). Recently, surgeons have shown success in treating non-idiopathic clubfoot with Ponseti casting (4–16).

Despite similar correction rates between STR and Ponseti casting, non-idiopathic clubfoot treatment with STR has shown unfavorable long-term results (17). Niki et al. reported a reoccurrence rate of 73% in the treatment of 41 non-idiopathic clubfoot with an average follow up of nearly 10 years. Recurrent deformity was subsequently treated with further casting and secondary operative procedures (18). These findings have been observed in other studies supporting the notion that STR treatment of non-idiopathic clubfoot leads to additional more invasive secondary operations (3, 4, 7, 10, 17–20). Kowalczyk and Felus observed an average of 1.4 procedures per foot following primary STR in their recurrent and uncorrected non-idiopathic clubfoot patients. These secondary surgeries include repeat STRs, talectomies, osteotomies, and salvage procedures (4). Results following treatment with Ponseti casting have shown similar initial correction and successful treatment of relapsed feet with further casting and percutaneous tenotomy. This has allowed for correction of the deformity with reduced surgical intervention and reduced surgical invasiveness when surgery is necessary for correction. Recurrent feet following Ponseti casting typically show some level of improvement requiring only minimally invasive surgery in order to obtain final correction (4).

It is unclear in the literature whether Ponseti casting of non-idiopathic clubfoot avoids excessive invasive surgery. Kowalczyk and Felus did determine revision rates in their study comparing treatment outcomes of STR vs. Ponseti, however their study focused solely on arthrogrypotic clubfoot (4). This study aims to add to the body of literature regarding non-idiopathic clubfoot, specifically reporting treatment outcomes of all neuromuscular and syndromic clubfoot following treatment by both modalities. We specifically want to assess whether Ponseti casting of non-idiopathic clubfoot can prevent additional and/or more extensive surgery. We examine the rates of less extensive extracapsular and more extensive intracapsular surgeries performed.

## Materials and Methods

An IRB-approved retrospective comparative study of patients treated for non-idiopathic clubfoot between 2000 and 2020 was conducted. Inclusion criteria for this study include initiation of treatment prior to the age of 2, diagnosis of clubfoot secondary to a neuromuscular or syndromic condition, and a minimum of 1-year follow-up. Patients were excluded from the study if they were not diagnosed with non-idiopathic clubfoot or if there was insufficient data in their medical charts.

Patients who met the inclusion criteria were subdivided into two groups depending on their initial method of treatment, operative primary treatment with limited or radical STR or conservative primary treatment with Ponseti casting. Prior to 2010, patients with non-idiopathic clubfoot were treated primarily with STR followed with more extensive revision surgery if relapse occurred. Beginning in 2010, a protocol at our institution was adopted to first treat conservatively with Ponseti casting and are treated with surgery only when they have failed at least one additional attempt with casting and bracing after recurrence. Three surgeons performed treatments on the neuromuscular clubfeet and adopted this protocol with initial Ponseti casting.

Once patients were placed into the STR group or Ponseti group, variables of interest were collected from patient charts including initial age at treatment onset, follow up length, heel cord tenotomy, reoccurrence of deformity, number of surgeries performed, type of surgeries performed, total anesthesia time, total surgery time, number of reoperations, reoperation anesthesia time, and reoperation surgery time. Data was analyzed using Mann-Whitney *U*-test for continuous variables.

Types of surgeries was defined as Extra-capsular procedures (EC) only or Extra-capsular and Intra-capsular (EC+IC). Extra-capsular surgeries include percutaneous achilles tenotomy, open tendo-achilles lengthening, limited soft tissue releases at the posteromedial hindfoot/ankle, and tendon transfers. Intra-capsular surgeries include comprehensive release typically involving a tibiotalar and subtalar capsulotomy, wedge osteotomies, talectomies, and Syme amputations.

The number of surgeries performed specifically refers to distinct surgical encounters that potentially entailed multiple procedures. Reoperation variables involved all surgeries performed after initial treatment following reoccurrence of the deformity.

## Results

Between January 2000 and June of 2019, 42 patients were treated for neuromuscular or syndromic clubfoot. Eight patients were lost to follow up and one patient died. The remaining 33 patients were treated initially surgically with STR or conservatively with Ponseti casting. All 33 patients had begun treatment prior to the age of 2 and have at least 1 year of follow up. Twenty-four patients (42 clubfeet) were treated with Ponseti casting and the other nine patients (15 clubfeet) were treated with STR. A breakdown of the underlying etiologies are listed in Tables 1, 2. From our patient study group the most common diagnosis of non-idiopathic clubfoot was attributed to spina bifida (45%) with the second most common being arthrogryposis (24%). Reoccurrence rates between the various etiologies were similar ranging from 50 to 87.5% and are listed in Table 3.

**Table 1:** Etiology of non-idiopathic clubfoot.

**Primary disorder**	**Ponseti group (*n* = 24 patients)**	**STR group (*n* = 9 patients)**
Arthrogryposis	6	2
Spina bifida	8	7
Chromosomal abnormality	4	0
Tethered spinal cord	1	0
Sacral agenesis	2	0
Other	3	0

**Table 2:** Patient demographics.

	**Ponseti**	**STR**	***p*-value**
Unilateral/bilateral	6/18	3/6	1.0
Age of initial Treatment	23	50.5	0.002
(weeks)			
Follow-up (months)	39.2	85.7	0.001
Gender	10 males, 14 females	2 males, 7 females	0.3

**Table 3:** Recurrence rate by etiology.

**Disorder**	**Recurrence rate**
Spina bifida	86.7% (13/15)
Arthrogryposis	87.5% (7/8)
Chromosomal abnormality	50% (2/4)
Other neuromuscular condition	50% (3/6)

### Ponseti Group

This subgroup is composed of 24 children (42 clubfeet) with 10 males and 14 females. The mean follow up was 39.2 months (range, 12–147 months) with mean initial age of treatment of 23 weeks (range, 2–62 weeks). The average number of casts was 4.7 per foot for initial correction (range, 1–9). Percutaneous achilles tenotomy was performed on all but one patient as part of the initial course of treatment. Following casting series and percutaneous achilles tenotomy, feet were placed in ankle foot orthotics (AFOs) in order to maintain correction. Recurrence of the deformity occurred in 71.4% of this subgroup. Reoccurrence was addressed primarily by repeat casting and achilles tenotomies. Patients who failed repeated casting or had severe reoccurrence of the deformity were treated surgically.

Among this cohort, 41 of the 42 clubfeet had surgeries with a total of 61 operations averaging at 1.45 operations per foot. EC procedures were performed in 97.6% of this cohort with only one patient not requiring a percutaneous achilles tenotomy. IC in addition to the EC procedures were required for 50% of this cohort with a majority being a comprehensive clubfoot release which typically consists of a posterior release; medial release; tibiotalar release; subtalar release; and flexor digitorum longus, flexor hallucis longus, and tendo-achilles lengthening. The average anesthesia and surgery time was 113.5 and 67.8 min, respectively. Reoperation rates included an average of 0.88 reoperations per foot, anesthesia time of 80.1 min, and surgery time of 50.8 min. No complications occurred following surgery in this cohort.

### STR Group

This subgroup is composed of 9 children (15 clubfeet) with 2 males and 7 females. The mean follow up was 85.7 months (range, 12–132) with mean initial age of treatment of 50.5 weeks (range, 8–104). This cohort was treated initially surgically with comprehensive posteromedial release. Following surgery, patients were placed into AFOs to maintain correction. Reoccurrence of the deformity occurred in 93.3% with only one patient maintaining correction following initial treatment.

Comprehensive posteromedial release was performed in all patients of this group with a total of 63 operations averaging at 4.2 per foot. Talectomies were the most common procedure performed following initial treatment and served more as a salvage operation. The average anesthesia and surgery time was 291.1 and 179.2 min, respectively. Reoperation rates included an average of 3.2 reoperations per foot, anesthesia time of 231.2 min, and surgery time of 138.6 min. Complication rate was 33% in this group with all three patients having post-surgical infections. Two patients underwent amputation due to recurrent ulceration, infection, and failed surgical correction.

Statistical analysis of the measured variables from both groups revealed significant findings as outlined in Tables 3, 4. The Mann-Whitney test showed significantly shorter anesthesia and surgery times in the Ponseti group compared to those of the STR group (*p* = 0.02, *p* = 0.01, respectively). The Mann-Whitney test found that the Ponseti group required significantly less surgery (*p* = 0.001) with almost 3 less operations per foot based on simple comparison of the means. With respect to the surgeries performed, the Mann-Whitney test indicated more invasive surgeries were performed in the STR group (*p* = 0.001) as suggested by a comparison of the EC+IC procedures performed in each group. When looking at reoperation rates following reoccurrence of the deformity, the Mann-Whitney test showed significantly shorter reoperation anesthesia and surgery time between the cohorts (*p* = 0.02, *p* = 0.03) and reduced number of reoperations (*p* = 0.01). The Mann-Whitney test did not determine significant differences in rate of recurrence (*p* = 0.085) and rate of EC procedures performed (*p* = 0.55) between Ponseti and STR groups.

**Table 4:** Descriptive statistics.

	**Ponseti**	**STR**	***Z*-test**	***p*-value**
Total anesthesia time (minutes)	113.5 (σ = 109.3)	291.1 (σ = 273.6)	−2.32	0.02
Total surgery time (minutes)	67.8 (σ = 66.4)	179.2 (σ = 161.7)	−2.57	0.01
Number of surgeries	1.45 (σ = 1.17)	4.2 (σ = 3.19)	−3.27	0.001
Reoperation anesthesia time	80.1 (σ = 99.4)	231.2 (σ = 265.1)	−2.33	0.02
Reoperation surgery time	50.8 (σ = 61)	138.6 (σ = 158.6)	−2.18	0.03
Number of reoperations	0.88 (σ = 1.02)	3.2 (σ = 3.19)	−2.51	0.01
Extracapsular	97.6%	100%	−0.598	0.55
Extracapsular and intracapsular	50%	100%	−3.416	0.001
Recurrence	71.4%	93.3%	−1.72	0.085

## Discussion

Non-idiopathic clubfoot has traditionally been viewed as resistant to non-operative treatment and is best addressed surgically. This is due to the severe deformity observed upon initial presentation that is typically difficult to correct, with persistent underlying soft tissue abnormalities predisposing to more rigid clubfoot deformity. Furthermore, neuromuscular and syndromic clubfoot patients often have a myriad of other ailments in addition to the foot deformity that require immediate attention. This can lead to delays in addressing the deformity often making correction more challenging.

Recently, this view has been challenged as several groups have reported favorable results when applying the Ponseti method to this patient population. Several studies have reported initial correction and reoccurrence rates of Ponseti casting on non-idiopathic clubfoot and these include Janicki et al. reporting initial correction of 90% and reoccurrence of 44%, Moroney et al. reporting initial correction of 90.7% and reoccurrence of 43.6%, Dunkley et al. reporting initial correction of 95.7% and reoccurrence of 36.4%, and Shah et al. reporting initial correction of 92.5% and reoccurrence of 42.5% (8–10, 16). Our experience with the Ponseti method has been complimentary to what's been reported in literature. Initial correction was obtained in all feet following casting and achilles tenotomy, and a higher recurrence rate (71.4%) was observed. The higher reoccurrence rate observed in our cohort can be attributed to poor compliance to the bracing protocol (47.6% non-compliant). Compliance was determined solely on parent's report with the brace. Despite the high rate of reoccurrence, these feet have responded well to recasting and repeat achilles tenotomy and have maintained correction of up to 54 months since initiation of treatment without the need of further intervention.

Soft tissue release is viewed as the more traditional approach in correcting non-idiopathic clubfoot. There are several studies available in literature that have reported outcomes regarding STR with non-idiopathic clubfoot however, a majority of them are disease specific with the most common being arthrogryposis and spina bifida. Arthrogryposis specific studies include Sodegard et al. who reports a recurrence rate of 20.8%, Niki et al. with a recurrence rate of 73%, and Kowalczyk and Felus with a recurrence rate of 64.2% (4, 18). Spina bifida specific studies include de Carvalho Neto et al. reported a recurrence rate of 37%, Flynn et al. with a recurrence rate of 39%, and Akbar et al. with a recurrence rate of 17% (21–23). Our experience has shown comparable results with a 78% recurrence however, our cohort is composed of multiple etiologies. Regardless, treatment was similar with initial soft tissue release followed by more invasive revision surgery for recurrent deformity. Our approach to correcting recurrent deformity in this cohort was consistent with other groups (4, 18, 21–23).

The purpose of this retrospective study was to evaluate Ponseti and STR in correcting non-idiopathic clubfoot, specifically examining whether Ponseti leads to less invasive surgery. We chose to evaluate this by characterizing our surgeries as either extra-capsular or intra-capsular, referring to whether the surgery involves the joint capsule of the talus. Analysis of our cohorts suggest Ponseti helps lead to sustained correction of the deformity in a manner that involves less invasive surgery. Specifically, we found that out of our cohort treated initially with Ponseti casting, only 50% required Intra-capsular surgical revision. This is significantly decreased relative to our STR group in which all were treated with intra-capsular procedures. This notion is additionally supported by significantly decreased surgery and anesthesia times as well as significantly decreased number of total surgeries per foot.

This has been a theme shared in several other studies (4, 9, 10). Mentioned previously by other groups, treating non-idiopathic clubfoot initially with Ponseti casting allows for partial correction of the deformity. This provides several benefits in that these patients are more likely to tolerate bracing, surgery (if necessary) can be delayed, and less invasive surgery is required due to the milder deformity (4, 9). This is important considering the poor outcomes that have been reported following surgical revision of relapsed feet with talectomies and radical STRs (24, 25). Legaspi et al. involved long-term results following revisional surgery with talectomies and reported only 8/24 feet (33%) sustaining full correction without complication or additional surgery (24). In our series, Talectomies were only performed in the STR group with 10/15 feet (67%) undergoing this procedure. Of these 10 feet, 7 had poor outcomes including recurrent ulceration, infection, and additional surgery.

This study has several limitations. Our cohorts were not strictly uniform as clubfeet were secondary to a number of etiologies as listed in Table 1. This can lead to discrepancies in clubfoot as different neuromuscular and syndromic disorders can lead to varying degrees of severity. The objective characterization and classification of deformity at presentation and after treatments were poorly documented, and documented recurrence and need for surgery were used as a proxy for significant recurrence. Furthermore, treatment was provided by three surgeons which can lead to some variability in treatment methods and surgical technique affecting outcomes. The differences in treatment initiation between the cohorts can be attributed to the differing protocols underlying each treatment method. In Ponseti casting, an effort was made to initiate casting as early as possible in order to maximize any potential flexibility in the feet and reduce the severity of the deformity. Conversely, STR's were performed traditionally after 6 months however this could change depending on the severity of the initial presentation. Lastly, follow-up length allowed for assessment of midterm results in the Ponseti group however, there is certainly a window for future reoccurrence of the deformity. Therefore, it is difficult to ascertain whether further revisional surgery is necessary for potential relapses.

In conclusion, the Ponseti method should serve as the primary approach in treating non-idiopathic clubfoot. Even though our patient population had high rate of recurrence, the protocol for casting provides several benefits worthy of the provider and patient's time investment. This data indicates the decreased need for complex, intra-capsular surgery. Also, this may benefit patients by reducing complication rates, need for numerous surgeries, as well as anesthesia and surgical times when surgical treatment of recurrence is undertaken. Improvement in patient function and these previously mentioned parameters are areas for future investigation.

## Data Availability Statement

The raw data supporting the conclusions of this article will be made available by the authors, without undue reservation.

## Ethics Statement

The studies involving human participants were reviewed and approved by TTUHSC Lubbock Institutional Review Board. Written informed consent from the participants' legal guardian/next of kin was not required to participate in this study in accordance with the national legislation and the institutional requirements.

## Author Contributions

JA, JW, and CB: study design. JA and JW: data acquisition and statistical analysis with interpretation of data. JA, JW, MD, and CB: drafting and revision of manuscript. All authors contributed to the article and approved the submitted version.

## Conflict of Interest

The authors declare that the research was conducted in the absence of any commercial or financial relationships that could be construed as a potential conflict of interest.
